# What Lies Beneath Trait-Anxiety? Testing the Self-Regulatory Executive Function Model of Vulnerability

**DOI:** 10.3389/fpsyg.2019.00122

**Published:** 2019-01-30

**Authors:** Henrik Nordahl, Odin Hjemdal, Roger Hagen, Hans M. Nordahl, Adrian Wells

**Affiliations:** ^1^Department of Psychology, Norwegian University of Science and Technology, Trondheim, Norway; ^2^Nidaros District Psychiatric Center, St. Olav’s University Hospital, Trondheim, Norway; ^3^Department of Mental Health, Norwegian University of Science and Technology, Trondheim, Norway; ^4^Division of Clinical and Health Psychology, School of Health Sciences, Faculty of Biology, Medicine and Health, Manchester Academic Health Science Centre, The University of Manchester, Manchester, United Kingdom; ^5^Greater Manchester Mental Health NHS Foundation Trust, Prestwich, United Kingdom

**Keywords:** metacognitive beliefs, trait-anxiety, risk factors, anxiety, depression, resilience

## Abstract

Vulnerability to psychological disorder can be assessed with constructs such as trait anxiety and neuroticism which among others are transdiagnostic risk factors. However, trait-anxiety and related concepts have been criticised because they don’t illuminate the etiological mechanisms of psychopathology. In contrast, the metacognitive (S-REF) model offers a framework in which metacognitive knowledge conceptualised in trait terms is part of a core mechanism underlying trait-anxiety and related constructs. The present study therefore set out to explore metacognitions as potential underlying factors in trait-anxiety (the propensity to depression and anxiety). Nine hundred and eighty two participants completed self-report measures of metacognitions and trait-anxiety at time 1, and 425 individuals completed the same measures 8 weeks later. At the cross-sectional level, metacognitions accounted for 83% of the variance in anxiety- and 64% of depression propensity. Furthermore, despite both domains of trait-anxiety showing high stability over time, negative- and positive metacognitive beliefs were significant prospective predictors of both domains of vulnerability. These findings suggests that metacognitive beliefs may be an underlying mechanism of vulnerability attributed to trait-anxiety with the implication that the metacognitive (S-REF) model informs conceptualization of psychological vulnerability, and that metacognitive therapy applications might be employed to enhance psychological resilience.

## Introduction

Founded in personality research, the concept of psychological vulnerability can be assessed by a variety of trait constructs such as Trait-Anxiety, Neuroticism and negative affectivity (Eysenck and Eysenck, 1975; Spielberger et al., 1983; Watson and Clark, 1984). These constructs are positively linked with psychopathology and are considered to be a general tendency to experience negative emotions that is genetically influenced (e.g., Rosenström et al., 2018). They are reliably associated with psychological disorders (Clark and Watson, 1991; Brown et al., 1998; Kotov et al., 2010; Mincic, 2015), broader aspects of physical health and illness, subjective well-being, relationship satisfaction, and social and occupational impairment (Lengel et al., 2016). It has been argued that trait theory has been underutilised in clinical settings (e.g., Barlow et al., 2014; Lengel et al., 2016; Watson et al., 2016), and that formulating and targeting traits such as negative affectivity could potentially advance our understanding of psychopathology (Sauer-Zavala et al., 2017).

One of the most frequently used measures of negative affectivity in psychological research is the State-Trait Anxiety Inventory (STAI: Spielberger et al., 1983). The STAI was designed by Spielberger et al. (1983) to measure anxiety as a state at a given point in time (state anxiety), and as a trait reflecting proneness to react with anxiety under stressful circumstances (trait anxiety). Trait anxiety is a dimension along which people vary, and can be invoked to explain individual differences in the frequency, intensity, and duration of episodes of state anxiety and negative affect. More recent studies employing factor analyses have suggested that the STAI-T consist of two inter-related factors and that its items measure propensity to both anxiety and depression (Bieling et al., 1998; Grös et al., 2007; Bados et al., 2010; Balsamo et al., 2013). Hence, rather than being considered a measure of specific proneness to anxiety as originally proposed, trait-anxiety should be considered a measure of general vulnerability to emotional disorder and distress.

Although the trait-anxiety construct has proven useful in the assessment of vulnerability and prediction of emotion disorder symptoms, critics have argued that personality dispositions such as negative affectivity or trait-anxiety do not yield useful information on the etiological mechanisms of psychopathology (Claridge and Davis, 2001; Ormel et al., 2004). Furthermore, the mechanisms underlying them must be elucidated in conceptualising these traits as central vulnerability factors (see e.g., Cuijpers et al., 2010; Ormel et al., 2013). One possibility is that there is overlap in vulnerability to both anxiety and depression and related constructs such as negative affect and these might be related to some common set of underlying psychological processes.

In the Self-Regulatory Executive Function (S-REF) model, Wells and Matthews (1994) argue that the differences between disorders are less important than the similarities, and that underlying transdiagnostic mechanisms of distress rather than topographical differences should become a greater focus in psychopathology research. In this approach, emotional disorders are viewed as caused by a common negative and perseverative thinking style, called the cognitive attentional syndrome (CAS; Wells, 2009). The CAS consist of worry and rumination, threat monitoring and maladaptive coping strategies that impair self-regulation. Furthermore, the CAS is regulated by underlying metacognitive beliefs conceptualised in trait terms, which includes knowledge about thinking, memory and attention (Wells and Matthews, 1994). Thus, metacognitive knowledge (i.e., metacognitive beliefs) are formulated as a central factor in both state and trait emotion, and might therefore be a core underlying mechanism in trait-anxiety and related constructs. For example, negative metacognitive beliefs about the uncontrollability and danger of worry in particular are likely to predict depression and anxiety proneness by contributing to reduced investment in controlling thinking and also to negative interpretations of internal experience, compromising choice of effective coping strategies when exposed to stress (Wells and Matthews, 1994).

Based on the S-REF model, there are two main measures which have been developed to assess generic metacognitive beliefs: the Metacognitions Questionnaire (MCQ; Cartwright-Hatton and Wells, 1997) and a briefer version, the Metacognitions Questionnaire-30 (Wells and Cartwright-Hatton, 2004). These trait measures consists of five factors assessing positive beliefs about worry, negative beliefs about uncontrollability and danger of worry, confidence in memory/attention, beliefs about the need to control thoughts, and cognitive self-consciousness. The five factor structure has been reported as reliable (Spada et al., 2008) and can account for individual variance in distress beyond a general “metacognition” factor (Fergus and Bardeen, 2017).

In line with predictions of the metacognitive model, metacognitive beliefs are demonstrated to be reliably associated with state measures of anxiety and depression (see Sun et al., 2017 for a review). In addition, significant positive correlations have been reported between metacognitive beliefs and trait-anxiety (Cartwright-Hatton and Wells, 1997; Wells and Cartwright-Hatton, 2004). One study has shown that metacognitive beliefs positively predicted trait-anxiety when controlling for the presence of a diagnosed mental disorder (Nordahl and Wells, 2017). Among domains of metacognitive beliefs, negative metacognitive beliefs have consistently shown the strongest association with trait-anxiety. However, to our knowledge no study has tested the structural relations between each domain of metacognitive belief and the two domains of trait-anxiety or explored these relations over time.

The aim of the current study was therefore to explore the association between the different domains of metacognitive beliefs and domains of trait-anxiety using both a cross-sectional and longitudinal data-set. To evaluate the structural relationship of these variables and test the overall fit of models, we employed structural equation modelling. Derived from the S-REF model (Wells and Matthews, 1994), our hypotheses were as follows; (1) metacognitive beliefs will be positively correlated with the STAI-T depression and anxiety factors; (2) metacognitive beliefs will explain substantial variance in both STAI-T factors; (3) metacognitive beliefs will account for variance in STAI-T factors over time; and (4) negative metacognitive beliefs will be the strongest independent predictor of both the STAI-T factors in the cross-sectional- and in the longitudinal data.

## Materials and Methods

### Participants and Procedure

The present study was based on an online self-report survey of psychological distress with two measuring points. The survey was conducted in Norway and was approved by the Regional Committee for Medical and Health Research Ethics (REC; reference: REK-Midt, 2016/705). Participants were invited to participate through advertisement on social media (Facebook), and were offered participation in a lottery to win an I-pad if they completed the survey at both time points. Several Norwegian voluntary organisations for mental health assisted in distributing information about the survey. Thus, participants were gathered at convenience, but had to be 18 years old or above, and had to able to read Norwegian. The survey was conducted using a programme called “Select Survey,” provided by the first author’s faculty at the Norwegian University of Science and Technology. Upon entering the survey portal, participants were presented with an information sheet that was approved by REC and were informed that proceeding to the main survey would be regarded as a signed informed consent. Nine hundred and eighty two individuals completed the metacognitions questionnaire 30 (MCQ-30; Wells and Cartwright-Hatton, 2004) and the State-Trait Anxiety Inventory; Trait version (STAI-T; Spielberger et al., 1983) at time 1 (T1), and four hundred and twenty five also completed the same measures at time 2 (T2), 8 weeks after the first round of questionnaires. The sample characteristics for the cross-sectional- and the longitudinal sample are presented in Table 1.

**Table 1:** Sample characteristics in the cross-sectional- and the longitudinal data sets.

	Cross-sectional	Longitudinal
	*n* = 982	*n* = 425
Age; mean (SD)	27.75 (9.49)	30.09 (9.84)
Female; n (%)	740 (75%)	344 (81%)
Single; n (%)	438 (45%)	162 (38%)
In a relationship; n (%)	167 (17%)	56 (13%)
Cohabitant/married; n (%)	336 (34%)	194 (46%)
Divorced; n (%)	38 (4%)	12 (3%)
Marital status missing; n	3	1
Working full time; n (%)	406 (41%)	144 (34%)
Students; n (%)	327 (33%)	145 (34%)
Working part time; n (%)	60 (6%)	34 (8%)
Unemployed; n (%)	24 (2%)	14 (3%)
Short term sick leave; n (%)	14 (1%)	10 (2%)
Long term sick leave; n (%)	119 (12%)	76 (18%)
Retired; n (%)	29 (3%)	1 (1%)
Occupational status missing; n	3	1


### Measures

#### The Metacognitions Questionnaire 30

The MCQ-30 (Wells and Cartwright-Hatton, 2004) is a 30-item self-report scale measuring beliefs about thinking (i.e., metacognitive beliefs). Each item are scored on a four-point scale ranging from 1 (do not agree) to 4 (agree very much), and each subscale has a range from 6 to 24 points. High scores reflect more reported problems with the construct in question. A five-factor structure exists: (1) positive beliefs about worry (e.g., “I need to worry in order to stay organised”); (2) negative beliefs about the uncontrollability and corresponding danger of worry (e.g., “my worrying thoughts persists, no matter how I try to stop them”); (3) cognitive confidence (e.g., “I do not trust my memory”); (4) beliefs about need to control thoughts (e.g., “I will be punished for thinking certain thoughts”); and (5) cognitive self-consciousness (e.g., “I am constantly aware of my thinking”). The measure has shown good internal consistency with Cronbach’s alpha ranging from 0.72 to 0.93 (Wells and Cartwright-Hatton, 2004) and has been validated in Norwegian samples (e.g., Grøtte et al., 2016). In the current study, the internal consistency was good (positive beliefs: α = 0.85, negative beliefs: α = 0.85, cognitive confidence: α = 0.88, need for control: α = 0.81, cognitive self-consciousness: α = 0.79).

#### The State-Trait Anxiety Inventory – Trait Scale

The State-Trait Anxiety Inventory (trait version: form Y2) (STAI-T: Spielberger et al., 1983) is a 20 item self-report questionnaire of general distress proneness, and has been validated in Norwegian samples (e.g., Haseth et al., 1990). Each item is rated on a four-point Likert scale. Total scores range from 20 to 80 points, with higher scores reflecting stronger traits of general distress proneness. The STAI-T has good psychometric properties, with Cronbach’s alpha in the range of 0.86 to 0.95, and test-retest correlations ranging from 0.73 to 0.86 (Spielberger et al., 1983). Further psychometric evaluation of the STAI-T has shown that it consists of two factors: (1) depression (e.g., “I feel like a failure”); and (2) anxiety (e.g., “I feel nervous and restless”). The depression factor consist of 13 items (item number; 1, 3–7, 10, 12–16, 19), while the anxiety factor consist of 7 items (item number; 2, 8–9, 11, 17–18, 20) (Bieling et al., 1998; Bados et al., 2010; Balsamo et al., 2013). The depression score ranges from 13 to 52 points, while the anxiety score ranges from 7 to 28 points. In the current study, the internal consistency was excellent (α = 0.96) for the total scale, and for the subscales; depression, α = 0.95; anxiety, α = 0.90.

### Statistical Analyses

Confirmatory factor analysis (CFA) was used to evaluate the factor structure of the proposed five-factor model of the MCQ-30 and the two-factor structure of the STAI-T. No secondary loadings were modelled, but the factors were allowed to inter-correlate. Bivariate correlations were used to explore the association between the MCQ-30- and the STAI-T subscales. Structural equation modelling was employed to evaluate the fit of an overall model were the MCQ-30 factors were used as predictors of the STAI-T factors in cross-sectional datasets. Three commonly recommended fit statistics were used to evaluate the models (Hu and Bentler, 1999; Kline, 2011; Brown, 2015); the comparative fit index (CFI), the standardised root mean square residual (SRMR) and root mean square error of approximation (RMSEA). The CFI should be above 0.90 to represent an adequate fit, the SRMR should be less than 0.08, and the RMSEA should be below or close to 0.06 and the upper limit of the 90% RMSEA confidence interval should not exceed 0.10. Finally, multiple hierarchical linear regression analyses were used to explore the prospective relationships between the MCQ-30 subscales and the STAI-T subscales.

## Results

### Factorial Structure of the MCQ-30 and the STAI-T

Initially we tested the 5 factor model of the MCQ-30 and the 2 factor model of the STAI-T using confirmatory factor analysis. In the T1 data, the MCQ-30 five factor measurement model showed the following fit indices: χ^2^(395) = 1622.05, *p* < 0.01, CFI = 0.90, SRMR = 0.07, RMSEA = 0.06 (90% CI = 0.05, -0.06), and in the T2 data, the fit indices were: χ^2^(395) = 1245.85, *p* < 0.01, CFI = 0.89, SRMR = 0.07, RMSEA = 0.07 (90% CI = 0.07, -0.08). The STAI-T two factor measurement model showed the following fit indices in the T1 data: χ^2^(169) = 961.63, *p* < 0.01, CFI = 0.93, SRMR = 0.04, RMSEA = 0.07 (90% CI = 0.07, -0.07), and χ^2^(169) = 714.12, *p* < 0.01, CFI = 0.92, SRMR = 0.05, RMSEA = 0.09 (90% CI = 0.08, -0.09) in the T2 data. Globally, these fit indices indicate an acceptable fit of the MCQ-30 five factor model and the STAI-T two factor model in this sample at T1 and at T2. Thus, we considered it acceptable to proceed with the planned analysis involving testing of relationships between multi-factorial constructs.

### Descriptive Statistics and Correlations Between Factors

As a first step, before testing predictive models, we ran correlational analyses to examine the basic pattern of relationships between domains of metacognitive beliefs and domains of trait-anxiety (i.e., depression and anxiety) in the data from T1. Descriptive statistics and bivariate correlations between measures are presented in Table 2. All of the correlations were positive and significant at the 0.01 level. STAI-T depression and anxiety were strongly correlated with each other, and showed the strongest correlation with negative metacognitive beliefs among the MCQ-30 subscales.

**Table 2:** Descriptive statistics and bivariate correlations among metacognitive- and trait-anxiety variables at time 1 (*N* = 982).

		2	3	4	5	6	7	M	SD
**1.**	MCQ-30pos	0.287^∗^	0.227^∗^	0.461^∗^	0.371^∗^	0.292^∗^	0.323^∗^	9.63	3.45
**2.**	MCQ-30neg		0.465^∗^	0.660^∗^	0.509^∗^	0.721^∗^	0.803^∗^	12.78	4.96
**3.**	MCQ-30cc			0.470^∗^	0.232^∗^	0.467^∗^	0.447^∗^	11.53	4.82
**4.**	MCQ-30nc				0.521^∗^	0.587^∗^	0.648^∗^	10.39	4.00
**5.**	MCQ-30csc					0.377^∗^	0.497^∗^	13.57	4.05
**6.**	STAI-Tdep						0.835^∗^	31.41	10.11
**7.**	STAI-Tanx							15.85	5.47


*^∗^p < 0.01, MCQ-30pos = positive beliefs about worry, MCQ-30neg = negative beliefs about the uncontrollability and corresponding danger of worry, MCQ-30cc = cognitive confidence, MCQ-30nc = beliefs about the need to control thoughts, MCQ-30csc = cognitive self-consciousness, STAI-Tdep = trait-anxiety depression subscale, STAI-Tanx = trait-anxiety anxiety subscale.*

### Cross-Sectional Relationships Between MCQ-30 Factors and Depression- and Anxiety Proneness

To explore if MCQ-30 factors would statistically predict depression and anxiety proneness we used structural equation modelling (e.g., Kline, 2011). The two trait-anxiety factors, depression and anxiety, were used as latent dependent variables indirectly measured by their respective items (reported in the methods section). The five MCQ-30 factors were defined as predictor variables measured by their respective six items per factor.

**FIGURE 1:**
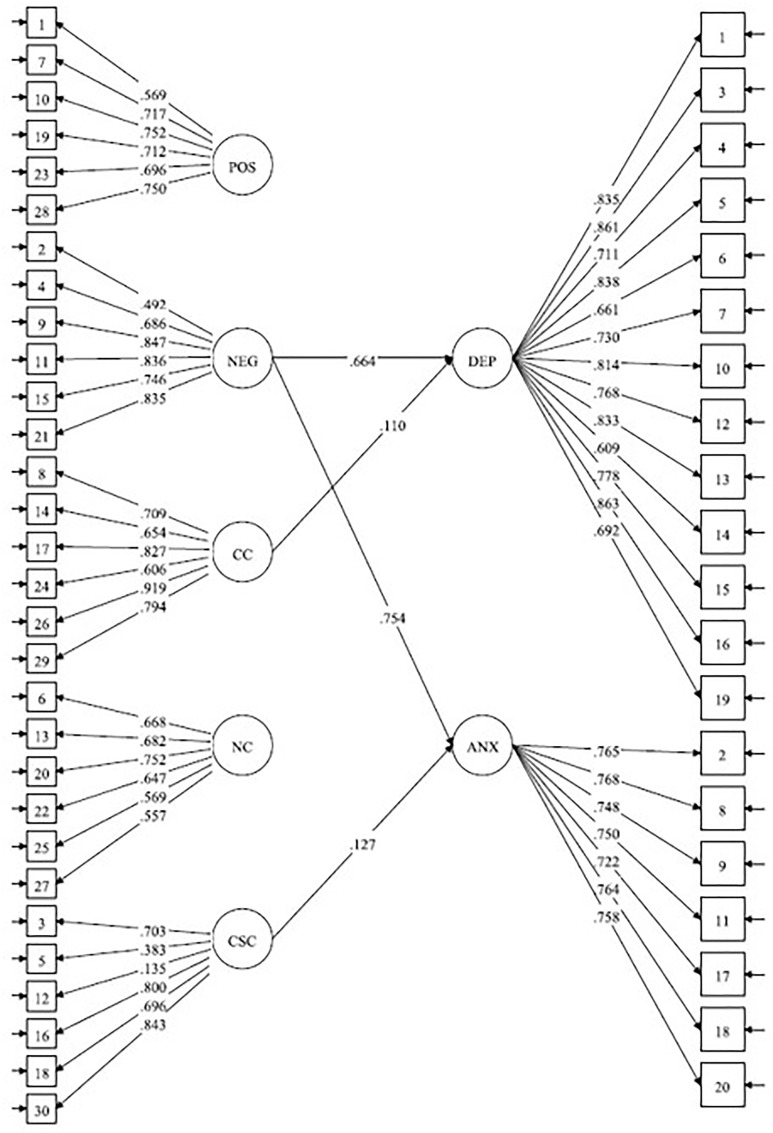
Structural equation model of the relationship between latent factors for the dimensions of the MCQ-30 and STAI-T. Ellipses represent latent variables, and rectangles represent observed variables (indicators). POS, positive beliefs about worry; NEG, beliefs about the uncontrollability and danger of worry; CC, cognitive confidence; NC, need for control; CSC, cognitive self-consciousness. DEP, STAI-T depression subscale; ANX, STAI-T anxiety subscale. The figure show standardised path coefficients and their significance at T1 (*N* = 982). Only significant lines are shown. Errors not shown; ^∗∗^*p* < 0.01 and ^∗^*p* < 0.05.

The hypothesised structural equation model is presented in Figure 1 and showed the following fit indices: χ^2^(1154) = 3604.10, *p* < 0.01, CFI = 0.91, SRMR = 0.06, RMSEA = 0.05 (90% CI = 0.05, -0.05), indicating an adequate model fit to the data. Moreover, 64% of the variance in STAI-T depression and 83% of the variance in STAI-T anxiety was explained by metacognitions in this cross-sectional model. Positive beliefs about worry and beliefs about the need to control thoughts did not account for a significant amount of variance in depression and anxiety. However, negative beliefs about the uncontrollability and corresponding danger of worry was found to predict a substantial proportion of the variance in both depression and anxiety and was the main predictor of both trait-anxiety constructs. Cognitive confidence was a significant predictor of depression, but not anxiety, and cognitive self-consciousness was a significant predictor of anxiety but not depression.

To determine the consistency of this cross-sectional model over time we re-ran it on the time 2 data. This model showed the following fit indices: χ^2^(1154) = 2723.25, *p* < 0.01, CFI = 0.90, SRMR = 0.07, RMSEA = 0.06 (90% CI = 0.05, -0.06), indicating an adequate model fit to the data. Moreover, 63% of the variance in STAI-T depression and 82% of the variance in STAI-T anxiety were explained by metacognitions in this model. Negative beliefs about the uncontrollability and corresponding danger of worry predicted both anxiety and depression and was the main predictor of both constructs. Cognitive confidence was also a significant predictor of both depression and anxiety. Cognitive self-consciousness was a significant predictor of anxiety, but not depression. The other MCQ-30 factors were not significant predictors of depression or anxiety in this model. Overall, the model from the T1 data was largely replicated in the T2 data, suggesting that the cross-sectional structural associations between constructs are consistent over time.

### Prospective Relationships Between MCQ-30 Factors and Trait Anxiety

To explore a potential causal association of metacognitions in trait-anxiety, we intended to run SEM with a two-wave cross lagged panel design, a method that has the potential to shed light on temporal precedence. However, the planned statistical approach could not be employed due to very high stability in both domains of trait-anxiety in the longitudinal data (*r* = 0.93, *p* < 0.001 for depression, and *r* = 0.87, *p* < 0.001 for anxiety), which potentially would lead to spurious cross-over effects (Kline, 2011). Thus, as an alternative we used hierarchical multiple regression analyses. First we ran two models where the trait-anxiety domains at T2 were used as dependent variables, and where gender/age, baseline symptom levels (T1 trait-anxiety; depression and anxiety) and T1 metacognitive belief domains where used as predictors. Gender/age, T1 depression/anxiety and T1 negative metacognitive beliefs were force-entered into the models, while forward entry was used for the remaining T1 metacognitive belief domains to explore if any of these domains entered the model when negative metacognitive beliefs were accounted for. Table 3 display results from these analyses.

**Table 3:** Statistics for the regression equations with time 2 STAI-T depression/anxiety as the dependent and metacognitive belief domains as predictors after controlling for gender/age and time 1 STAI-T depression/anxiety (*n* = 425).

	Step		*F* cha	*R*^2^ cha	β	*T*
**T2 Depression**						
	1		7.600	0.035**		
		Gender			0.18	3.890**
		Age			0.03	0.549
	2		2613.966	0.831**		
		Gender			-0.02	-1.184
		Age			-0.02	-0.838
		T1 Depression			0.94	51.127**
	3		10.808	0.003**		
		Gender			-0.03	-1.463
		Age			-0.01	-0.598
		T1 Depression			0.88	34.361**
		T1 MCQ-30neg			0.08	3.288**
	4		4.566	0.001*		
		Gender			-0.03	-1.586
		Age			-0.01	-0.324
		T1 Depression			0.87	34.043**
		T1 MCQ-30neg			0.08	3.086**
		T1 MCQ-30pos			0.04	2.137*
**T2 Anxiety**						
	1		14.398	0.064**		
		Gender			0.24	5.057**
		Age			-0.07	-1.421
	2		1182.145	0.690**		
		Gender			0.01	0.351
		Age			-0.01	-0.309
		T1 Anxiety			0.87	34.382**
	3		9.711	0.006**		
		Gender			0.01	0.365
		Age			-0.01	-0.501
		T1 Anxiety			0.76	18.066**
		T1 MCQ-30neg			0.13	3.116**
	4		4.595	0.003*		
		Gender			0.01	0.269
		Age			-0.01	-0.273
		T1 Anxiety			0.75	17.699**
		T1 MCQ-30neg			0.13	3.045**
		T1 MCQ-30pos			0.05	2.144*


*^∗^p < 0.05 and ^∗∗^p < 0.01 MCQ-30pos = positive beliefs about worry, MCQ-30neg = negative beliefs about the uncontrollability and corresponding danger of worry.*

In the final equations, negative- and positive metacognitive beliefs were significant predictors of both STAI-T depression and STAI-T anxiety measured 2 months later. The amount of variance accounted for by metacognitions was very small, a factor that is likely to result from the small amount of residual variance after controlling for time 1 trait-anxiety which changed little over time.

To further explore these findings, and shed some light on the directionality of associations between metacognitions and trait anxiety we ran two more hierarchical linear regressions where T2 MCQ-30 negative metacognitive beliefs, and T2 MCQ-30 positive metacognitive beliefs were used as dependent variables. In these models, we entered gender/age in the first step, T1 MCQ-30 negative-/positive metacognitive beliefs in the second step, and T1 STAI-T depression and STAI-T anxiety in the final step to explore whether T1 trait-anxiety domains could account for T2 metacognitions when T1 metacognitions were controlled. The results from these regressions suggested that STAI-T depression at T1 was not a significant predictor of T2 metacognitions. Moreover, T1 STAI-T anxiety was not a significant predictor of T2 positive metacognitive beliefs, but it did significantly predict T2 negative metacognitive beliefs.

## Discussion

This study aimed to examine domains of metacognitive beliefs as predictors of trait-anxiety, a marker of psychological vulnerability to depression and anxiety.

In the cross-sectional analyses we found that metacognitive beliefs were positively and significantly correlated with both trait-anxiety dimensions. Structural equation modelling of predictors of trait-anxiety domains showed an acceptable fit to the data with 64% of the variance in propensity to depression and 83% of the variance in propensity to anxiety explained by metacognitive beliefs. Here negative metacognitive beliefs were the most substantial contributor to both anxiety and depression with small additional contributions to anxiety of cognitive self-consciousness and to depression of cognitive confidence. The model was replicated in the cross sectional data at time 2 where an additional contribution of cognitive confidence to anxiety also emerged, but the overall model retained a good fit and showed stability of structural relations across time.

Longitudinal analysis informs the possible temporal relations between metacognition and psychological vulnerability. Here, we observed in the hierarchical regression that negative- and positive metacognitive beliefs prospectively predicted both domains of trait-anxiety of which negative metacognitive beliefs explained most of the individual variance. In the reverse model we found that STAI anxiety prospectively predicted negative metacognitive beliefs suggesting a bidirectional causal relationship between these constructs. However, for positive beliefs the pattern was uni-directional with positive beliefs at time 1 predicting both domains of trait-anxiety at time 2 but not the converse. Nonetheless, these results must be considered to be preliminary as other unmeasured factors may account for the relationships observed. Our results indicate a possible causal role for metacognitions in trait-anxiety, but the directionality in these factors requires more rigorous analysis.

The results from our study bring further support for the metacognitive model of psychological disorder, and question the concept of trait-anxiety as a core (indivisible) vulnerability factor. In the metacognitive perspective (Wells and Matthews, 1994; Wells, 2009), negative affectivity and related constructs such as trait-anxiety and neuroticism may be better understood as markers of maladaptive metacognitions and thinking styles [i.e., the cognitive attentional syndrome (CAS); Wells, 2009]. In the S-REF model, traits are mainly associated with metacognitive beliefs and self-knowledge, and states with the immediate extent and character of metacognitive strategies, namely the CAS (Wells and Matthews, 1994). Metacognitive beliefs (traits) and metacognitive strategies (states) are likely to interact such that maladaptive aspects of personality are enhanced by higher levels of CAS activation. The present data suggest bi-directionality of anxiety and specific negative metacognitions over time, with uni-directionality associated more with positive metacognitions. Trait anxiety may be a topological marker for both the activation of the CAS (e.g., worry/rumination) and of metacognitive beliefs that promote and maintain such processes.

Moreover, our findings confirm a central tenet of the metacognitive (S-REF) model; that both common (i.e., negative beliefs about uncontrollability and danger) and more specific domains of metacognitive beliefs can underlie different presentations of distress or vulnerability. Furthermore, different domains of metacognitions may serve as causal factors constituting vulnerability (i.e., negative- and positive metacognitive beliefs) and as maintenance factors (i.e., negative metacognitive beliefs, cognitive confidence and cognitive self-consciousness). The pattern of metacognitive predictors is interesting because negative beliefs about uncontrollability and danger emerged as a possible cause and consequence of trait-anxiety, which might be consistent with it having both a generative and maintenance role in susceptibility to distress.

The findings from the present study indicate that psychological vulnerability can be conceptualised within the S-REF model as predicted, a finding that has several clinical implications. Psychological vulnerability in the form of metacognitive knowledge can effectively be modified with Metacognitive therapy (MCT; Wells, 2009). A recent systematic review and meta-analysis shows that MCT is a highly effective treatment for anxiety and depression, and also that it effectively modifies maladaptive metacognitions (Normann and Morina, 2018). Several studies on MCT for individuals with generalised anxiety disorder have shown that severity of trait-anxiety decreases following treatment (Wells and King, 2006; Wells et al., 2010; van der Heiden et al., 2012; van der Heiden et al., 2013; Nordahl et al., 2018). Moreover, the S-REF model may inform further research on preventative mental health interventions. For example, it has been suggested that metacognitive therapy applications such as the Attention Training Technique (ATT; Wells, 1990, 2000) could enhance self-regulatory abilities in children by increasing flexible control over attention and thus modify maladaptive meta-level processes and knowledge (Murray et al., 2016, 2018).

This study has several limitations that should be acknowledged. First, the study relied on self-report measures, and a substantial proportion of the participants did not complete measures at time 2. Participants were mostly females. In addition, the sample was gathered at convenience online using social media, which may have biassed the sample characteristics (Wright, 2005). We must be cautious in generalising from these findings. Moreover, we had no control over current health status, meaning that some of the participants may have had psychiatric disorders and be experiencing levels of distress. Because of high stability in domains of trait-anxiety over 8 weeks, one should be cautious when drawing conclusions about the direction of causality based on this data. It remains to be determined if metacognitive belief domains also emerge as significant predictors of other measures of vulnerability such as neuroticism.

## Conclusion

In conclusion, the current study suggests that metacognitive beliefs may be an underlying mechanism of vulnerability attributed to trait-anxiety, and that there are both common and more specific domains of metacognitive beliefs associated with the propensity to depression and anxiety. This implies that “vulnerability” may be conceptualised within the metacognitive model and modified with metacognitive therapy (Wells, 2009) with a view to targeting specific dimensions of metacognitive knowledge and thus enhancing psychological resilience.

## Author Contributions

HN and AW planned the study and wrote a first draught of the manuscript and all authors contributed substantially to the finalised version. HN, OH, and RH carried out the survey. HN, OH, HMN, and AW conducted the data analyses.

## Conflict of Interest Statement

The authors declare that the research was conducted in the absence of any commercial or financial relationships that could be construed as a potential conflict of interest.
